# Biophysical models applied to dementia patients reveal links between geographical origin, gender, disease duration, and loss of neural inhibition

**DOI:** 10.1186/s13195-024-01449-0

**Published:** 2024-04-11

**Authors:** Sebastian Moguilner, Rubén Herzog, Yonatan Sanz Perl, Vicente Medel, Josefina Cruzat, Carlos Coronel, Morten Kringelbach, Gustavo Deco, Agustín Ibáñez, Enzo Tagliazucchi

**Affiliations:** 1 Latin American Brain Health (BrainLat), Universidad Adolfo Ibáñez, Av. Diag. Las Torres 2640, Santiago Región Metropolitana, Peñalolén, 7941169 Chile; 2 Global Brain Health Institute (GBHI), University of California San Francisco (UCSF), 1207 1651 4th St, 3rd Floor, San Francisco, CA 94143 USA; 3 Cognitive Neuroscience Center (CNC), Universidad de San Andrés, Vito Dumas 284, B1644BID Buenos Aires, VIC Argentina; 4 Department of Neurology, Massachusetts General Hospital and Harvard Medical School, 25 Shattuck St, Boston, MA 02115 USA; 5 National Scientific and Technical Research Council (CONICET), Godoy Cruz 2290, CABA, 1425 Argentina; 6 Institute of Applied and Interdisciplinary Physics and Department of Physics, University of Buenos Aires, Pabellón 1, Ciudad Universitaria, CABA, 1428 Argentina; 7 Center for Brain and Cognition, Computational Neuroscience Group, Universitat Pompeu Fabra, Plaça de La Mercè, 10-12, Barcelona, 08002 Spain; 8 Centro Interdisciplinario de Neurociencia de Valparaíso (CINV), Universidad de Valparaíso, Harrington 287, Valparaíso, 2381850 Chile; 9 Centre for Eudaimonia and Human Flourishing, Linacre College, University of Oxford, St.Cross Rd, Oxford, OX1 3JA UK; 10 Department of Psychiatry, University of Oxford, Warneford Hospital, Warneford Ln, Headington, Oxford, OX3 7JX UK; 11 Center for Music in the Brain, Department of Clinical Medicine, Aarhus University, Palle Juul-Jensens Blvd. 82, Aarhus, 8200 Denmark; 12 Department of Neuropsychology, Max Planck Institute for Human Cognitive and Brain Sciences, Stephanstraße 1a, Leipzig, 04103 Germany; 13 Institució Catalana de Recerca I Estudis Avancats (ICREA), Passeig de Lluís Companys, 23, Barcelona, 08010 Spain; 14 Turner Institute for Brain and Mental Health, Monash University, 770 Blackburn Rd,, Clayton, VIC 3168 Australia; 15 Trinity College Institute of Neuroscience, Trinity College Dublin, 152 - 160 Pearse St, Dublin, D02 R590 Ireland; 16 Trinity College Dublin, Lloyd Building Trinity College Dublin, Dublin, D02 PN40 Ireland

**Keywords:** Dementia, Neurodegeneration, Biophysical modeling, Hyperexcitability, Variability, Gender, Heterogeneity

## Abstract

**Background:**

The hypothesis of decreased neural inhibition in dementia has been sparsely studied in functional magnetic resonance imaging (fMRI) data across patients with different dementia subtypes, and the role of social and demographic heterogeneities on this hypothesis remains to be addressed.

**Methods:**

We inferred regional inhibition by fitting a biophysical whole-brain model (dynamic mean field model with realistic inter-areal connectivity) to fMRI data from 414 participants, including patients with Alzheimer’s disease, behavioral variant frontotemporal dementia, and controls. We then investigated the effect of disease condition, and demographic and clinical variables on the local inhibitory feedback, a variable related to the maintenance of balanced neural excitation/inhibition.

**Results:**

Decreased local inhibitory feedback was inferred from the biophysical modeling results in dementia patients, specific to brain areas presenting neurodegeneration. This loss of local inhibition correlated positively with years with disease, and showed differences regarding the gender and geographical origin of the patients. The model correctly reproduced known disease-related changes in functional connectivity.

**Conclusions:**

Results suggest a critical link between abnormal neural and circuit-level excitability levels, the loss of grey matter observed in dementia, and the reorganization of functional connectivity, while highlighting the sensitivity of the underlying biophysical mechanism to demographic and clinical heterogeneities in the patient population.

**Supplementary Information:**

The online version contains supplementary material available at 10.1186/s13195-024-01449-0.

## Background

The increasing prevalence and underdiagnosis of dementia represent a global challenge, which is more accentuated in diverse and non-stereotypical populations [1]. In comparison with other regions, individuals from Latin America (Latam) present larger genetic and socioeconomic heterogeneity [2] and they are also underrepresented in the scientific literature [3]. Biomarkers developed in high income countries (HIC) usually fail to generalize to Latam [4, 5], which could be explained by sample heterogeneity, where brain-phenotype models of non-stereotypical samples fail to provide reproducible results [6]. More robust and mechanistically-oriented computational models are required to characterize the biological underpinnings of population heterogeneity; crucially, robust and specific computational models for neurodegenerative diseases such as Alzheimer’s disease (AD) and frontotemporal dementia (FTD) are needed to understand the overlap and differentiation across brain phenotypes. While previous studies have applied whole-brain models to investigate AD [7–10], to date no systematic brain modelling comparisons have been developed to test model reproducibility and to address the effects of population heterogeneity associated with gender-specific effects.

Recent advances in neuroimaging-informed whole-brain models have enabled the investigation of pathophysiological mechanisms preceding neurodegeneration, especially those related with departures from excitation/inhibition (E/I) balance of neural networks [11]. A leading hypothesis suggests that these imbalances may lead to functional connectivity impairments associated with tau and amyloid β accumulation [12, 13]. Notably, epileptiform discharges and seizures occur in at least 20–40% of individuals with AD [14], many of which are undetected with current diagnostic procedures. Epileptogenic neuronal activity can increase both amyloid β and tau secretion, establishing a vicious cycle augmenting the aberrant aggregation and spread of the misfolding of these proteins [15]. Although these markers are less frequent in behavioral variant FTD (bvFTD) [16], they may be also present as non-convulsive seizures [17]. The prevalence of neural excitability over inhibition is supported by transgenic mouse models of AD [18], as well as by human studies probing cortical excitability using Transcranial Magnetic Stimulation (TMS) [19], and by the analysis of spontaneous activity recorded in vivo with MEG, establishing a shift towards higher excitation in a group of advanced AD patients with dementia but not in early-stage patients [20]. Another study also reported network-specific decreases in neural inhibition in AD patients [21]. Considered as a whole, these results support the hypothesis of impaired inhibition as a main contributor to AD pathophysiology.

An important limitation of the previous literature is the lack of studies conducted on non-stereotypical phenotypes. In particular, computational models assessing changes in excitation and/or inhibition in AD and bvFTD patients lack validation in underrepresented samples, such as those from Latin American countries. Conducting studies in these samples is important to determine how disease mechanisms are influenced by environmental, socioeconomic, and genetic factors. Moreover, even though the dynamics of the cerebral cortex are highly heterogeneous [22], few modeling studies to date aimed to understand how brain-regional structural heterogeneities (e.g., atrophy) impact on neural excitation and inhibition [23] as estimated using functional magnetic resonance imaging (fMRI), and how this can be mediated by geographical heterogeneity, disease duration, and demographic information.

In contrast to electrophysiological data, where the excitation/inhibition balance can be inferred from the power exponent of spontaneous activity [24], the estimation of local inhibition and/or excitation from fMRI signals should be informed by a model of the underlying neural populations. Therefore, we adopted a whole-brain semiempirical approach incorporating on subject-specific resting-state activity, group-level (AD and bvFTD) atrophy, and a diffusion tensor imaging (DTI) structural connectome, all combined to fit a dynamic mean field (DMF) model to the empirical functional connectivity [25]. Following this approach, we aimed to determine whether AD and bvFTD were associated with impaired local inhibition from resting state fMRI recordings. For this purpose, we chose to tune the local feedback inhibition control (FIC) parameter informed by local atrophy estimates. This choice is based on reports of synapse loss during early neurodegeneration [26, 27], which could affect the homeostatic plasticity necessary for the balance of excitatory and inhibitory inputs [28]. Based on previous results, we expected that decreases in the optimal inhibitory FIC parameter would occur when fitting the model to AD and bvFTD functional connectivity [29]. Moreover, we expected that incorporating disease-specific atrophy maps would optimize the fit to the functional data of each group, as previously reported in modeling studies of neurodegeneration [30], as well as in other datasets [25]. Given the more heterogeneous nature of disease presentation and progression in the Latam cohort due to higher variations in genetic factors and socioeconomic disparities [31, 32], we investigated whether diminished goodness of fit of the model could be observed in this dataset, relative to the less variable HIC cohort. We also investigated the potential effects of disease duration on loss of feedback inhibitory current, expecting a relationship between both variables in case the model reveals that loss of local inhibition is associated with neurodegeneration in AD and bvFTD. We tested for the possibility of gender differences between Latam and HIC, given the larger gender disparities that are measured in less developed countries, with potential repercussions on neurodegenerative processes [33, 34]. Finally, we expected that that whole-brain model would reflect disease-specific profiles of functional brain connectivity loss, with posterior nodes being more affected in AD and frontal nodes in FTD [35–37].

## Methods

### Participants

**Fig. 1:**
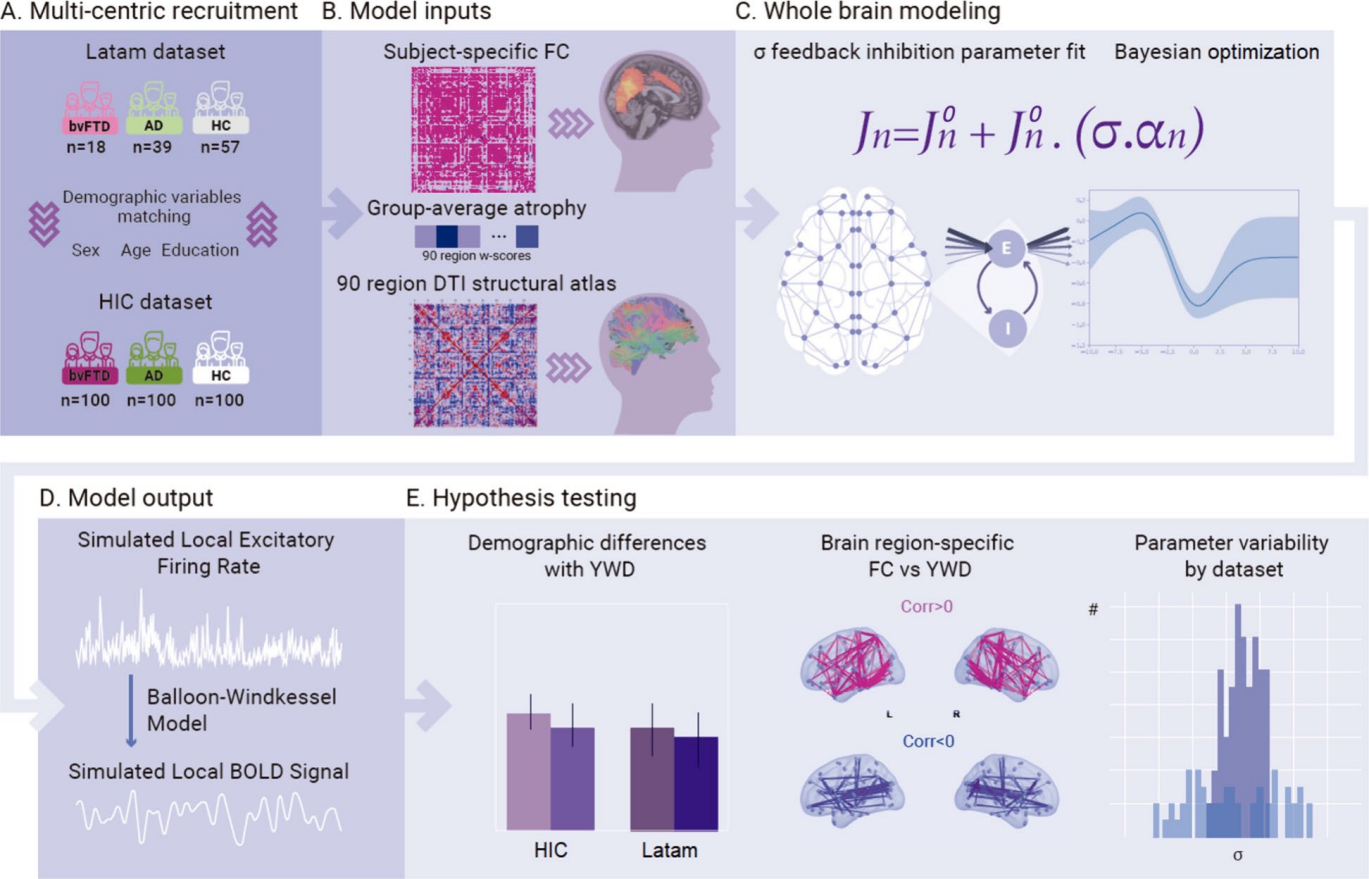
Methodological overview. **A** Recruitment from three centers in the Latam database consisted of demographically matched 18 bvFTD patients, 39 AD patients, and 57 HCs, while 100 bvFTD patients, 100 AD patients, and 100 HCs were downloaded to obtain a demographically matched HIC dataset. **B** Model inputs consisted of subject-specific FC in the AAL-90 atlas, group averaged atrophy patterns as 90 w-scores, and a 90 region DTI structural atlas. **C** Whole-brain modeling scheme fitting the $\sigma$ feedback inhibition parameter that multiplies the atrophy vector in the feedback inhibition current ${J}_{n}$ equation, tuned by Bayesian optimization (**D**) Model output consisting of simulated BOLD signals transformed from the excitatory firing rates using the Balloon-Windkessel model. **E** Hypothesis testing including the evaluation of the $\sigma$ parameter, associations with YWD and sex, brain region-specific FC changes with YWD, and $\sigma$ parameter variability analysis across datasets. FC: functional connectivity. YWD: years with disease; AD: Alzheimer’s disease. bvFTD: behavioral variant frontotemporal dementia. HC: healthy controls. DTI: diffusion tensor imaging

The sample consisted of two datasets from different regions, adding up to a total of 414 participants with MRI scans in two modalities: T1-weighted MRI and resting-state fMRI scans (*n* = 118 individuals with bvFTD, *n* = 139 individuals with AD, and *n* = 157 HC), matched for demographic variables (i.e., age, sex, and education) and by region (Table S1). Of the 414 participants, 18 individuals with bvFTD, 39 with AD, and 57 HC were obtained from samples of a pre-existing Latin America and the Caribbean (LAC) database, referred hereafter as the Latam sample (Table S1). The rest of the participants, 100 individuals with bvFTD, 100 with AD, and 100 HC were obtained from the Alzheimer’s Disease Neuroimaging Initiative (ADNI) (AD = 100 and HC = 50), the Neuroimaging in Frontotemporal Dementia (NIFD/LONI) (bvFTD = 100 and HC = 50), referred hereafter as the High Income Country (HIC) sample [38] (Table S1). Within the HIC sample we selected a subsample matching the Latam sample size (18 individuals with bvFTD, 39 with AD, and 57 HC), balanced through demographic variables (see Table S1) to run a reproducibility analysis. The Latam sample included participants that are underrepresented in the scientific literature and was obtained from the Multi-Partner Consortium to Expand Dementia Research in Latin America (ReDLat) [39] (Fig. 1A).

Across samples, the clinical diagnoses were produced by experts in dementia through an extensive neurological and neuropsychiatric examination comprising semi-structured interviews and standardized tests, with current criteria for probable bvFTD, and the National Institute of Neurological and Communicative Diseases and Stroke/Alzheimer’s Disease and Related Disorders Association (NINCDS-ADRDA) clinical criteria for AD [40, 41]. The patients did not present any psychiatric, vascular, or other neurological disorders. The inclusion of healthy control subjects required the confirmation of normal cognitive function, the absence of any disease, and an MRI structural scan free of lesions or significant white matter/atrophy changes. The IRB of each institution that contributed with MRI images to this study approved the acquisitions, and all the participants of this study signed a consent form following the declaration of Helsinki. The methods were performed in accordance with the guidelines and regulations and approved by the committee of the ReDLat Multi-Partner Consortium members [39].

### Neuroimaging acquisition and preprocessing

We obtained 3D structural volumetric and 10-min-long resting state fMRI sequences from all participants – the recordings were performed in three scanners for the Latam database, while for the HIC database, different scanners were used on each database but each group shared the same acquisition parameters (see Table S2 for details**)**.

### Whole-brain dynamic mean field modeling

Having the subject-specific empirical fMRI timeseries as inputs, together with the atrophy w-scores per subject group (obtained by subtracting the mean and dividing by the standard deviation of the healthy controls) [42] and a DTI connectome of structural connectivity employed in previous work from our group [30], we fitted a dynamic mean field (DMF) [25] model to reproduce the whole-brain FC (Fig. 1B). We then evaluated whether the model parameters inferred from the optimal fit correlated with demographic variables such as patients’ years with disease (YWD) and gender. The proposed model is a variant of that employed by Deco et al. (2018) [25], where the heterogeneity of the local feedback inhibition parameter is modulated by the atrophy map specific to each patient group, thus adjusting the local excitatory/inhibitory balance. The model comprises the 90 AAL-90 [43] nodes identified across cortical and subcortical regions (see Fig. 1C for a diagram of this approach). Each node consists of an excitatory neural population with local recurrent connections and long-range connections, and an inhibitory neural population having only local connections. All the model parameters are compatible with known empirical values [44], and result in simulated neural dynamics that are qualitatively similar to those observed in experimental data [45, 46].

Considering the n-th node, the equations for the excitatory ${{\text{I}}}_{{\text{n}}}^{\left({\text{E}}\right)}$ and inhibitory ${{\text{I}}}_{{\text{n}}}^{({\text{I}})}$ currents of the respective neural populations are the following:

1 $$\begin{array}{c}{{\text{I}}}_{{\text{n}}}^{\left({\text{E}}\right)}={{\text{W}}}_{{\text{E}}}{{\text{I}}}_{0}+{{\text{W}}}_{+}{{\text{J}}}_{{\text{NMDA}}}{{\text{S}}}_{{\text{n}}}^{\left({\text{E}}\right)}+G {{\text{J}}}_{{\text{NMDA}}}{\sum }_{{\text{p}}}{{\text{C}}}_{{\text{np}}}{{\text{S}}}_{{\text{p}}}^{\left({\text{E}}\right)}-{{\text{J}}}_{{\text{n}}}{{\text{S}}}_{{\text{n}}}^{\left({\text{I}}\right)}\\ {{\text{I}}}_{{\text{n}}}^{({\text{I}})}={{\text{W}}}_{{\text{I}}}{{\text{I}}}_{0}+{{\text{J}}}_{{\text{NMDA}}}{{\text{S}}}_{{\text{n}}}^{\left({\text{E}}\right)}-{{\text{S}}}_{{\text{n}}}^{({\text{I}})}\end{array}$$

With ${{\text{I}}}_{0}=0.382\mathrm{\ nA}$ as the external current, an excitatory scaling factor for ${{\text{I}}}_{0}$ of ${{\text{W}}}_{{\text{E}}}=1$, and an inhibitory scaling factor for ${{\text{I}}}_{0}$ of ${{\text{W}}}_{{\text{I}}}=0.7$. The excitatory glutamatergic synaptic coupling (mediated by N-methyl-D-aspartate receptors, [NMDA]), ${{\text{S}}}_{{\text{n}}}^{({\text{E}})}$, is associated with the current ${{\text{J}}}_{{\text{NMDA}}}=0.15\mathrm{\ nA}$, with a local excitatory recurrence weight of ${{\text{W}}}_{+}=1.4$, and a global coupling parameter ${\text{G}}$ = 2.5 [25] as a scaling factor for the structural connectivity given by ${{\text{C}}}_{{\text{np}}}$. The components of this matrix are determined by a DTI atlas [30]. The FIC parameter for region n, ${{\text{J}}}_{{\text{n}}}$, is defined as:

2 $${J}_{n}={J}_{n}^{0}(1+\sigma .{\alpha }_{n})$$

with ${\mathrm{\alpha }}_{{\text{n}}}$ given by the atrophy w-score of region n, obtained for each group separately (i.e., AD and bvFTD) as well as for each database (i.e., HIC and Latam). Here, the parameter $\upsigma$ represents the extent to which the local atrophy values modulate the baseline FIC parameter, ${{\text{J}}}_{{\text{n}}}^{0}$, which is optimized through recursive adjustments to clamp the firing rate within a neurobiologically plausible range of 3–4 Hz, following Deco et al. (2018) [25]. Thus, $\upsigma =0$ corresponds to the case when the atrophy does not influence the local inhibition, while its positive/negative influence is indicated by the sign of $\upsigma$.

The equations that define the firing rate of the excitatory ${{\text{r}}}_{{\text{n}}}^{({\text{E}})}$ and inhibitory neurons ${{\text{r}}}_{{\text{n}}}^{({\text{I}})}$ are defined as:

3 $$\begin{array}{c}{{\text{r}}}_{{\text{n}}}^{({\text{E}})}={{\text{H}}}^{({\text{E}})}\left({{\text{I}}}_{{\text{n}}}^{({\text{E}})}\right)=\frac{{{\text{g}}}_{{\text{E}}}\left({{\text{I}}}_{{\text{n}}}^{({\text{E}})}-{{\text{I}}}_{{\text{thr}}}^{({\text{E}})}\right)}{1-{\text{exp}}\left(-{{\text{d}}}_{{\text{E}}}{{\text{g}}}_{{\text{E}}}\left({{\text{I}}}_{{\text{n}}}^{({\text{E}})}-{{\text{I}}}_{{\text{thr}}}^{({\text{E}})}\right)\right)}\\ {{\text{r}}}_{{\text{n}}}^{({\text{I}})}={{\text{H}}}^{({\text{I}})}\left({{\text{I}}}_{{\text{n}}}^{({\text{I}})}\right)=\frac{{{\text{g}}}_{{\text{I}}}\left({{\text{I}}}_{{\text{n}}}^{({\text{I}})}-{{\text{I}}}_{{\text{thr}}}^{({\text{I}})}\right)}{1-{\text{exp}}\left(-{{\text{d}}}_{{\text{I}}}{{\text{g}}}_{{\text{I}}}\left({{\text{I}}}_{{\text{n}}}^{({\text{I}})}-{{\text{I}}}_{{\text{thr}}}^{({\text{I}})}\right)\right)}\end{array}$$

The gain factor of the excitatory ${{\text{H}}}^{({\text{E}})}$ and inhibitory ${{\text{H}}}^{({\text{I}})}$ neuronal responses transforms the currents according to Deco et al., 2018 [25]. The corresponding parameters are the conductance values, ${{\text{g}}}_{{\text{E}}}=310\ {{\text{nC}}}^{-1}$ and${{\text{g}}}_{{\text{I}}}=310\ {{\text{nC}}}^{-1}$, the threshold currents, ${{\text{I}}}_{{\text{thr}}}^{({\text{E}})}=0.403\ {\text{nA}}$ and ${{\text{I}}}_{{\text{thr}}}^{({\text{I}})}=0.288\ {\text{nA}}$, and constants determining the shape of the sigmoid function, ${{\text{d}}}_{{\text{E}}}=0.16$ and ${{\text{d}}}_{{\text{I}}}=0.087$. Finally, the excitatory, ${{\text{S}}}_{{\text{n}}}^{({\text{E}})}$, and inhibitory, ${{\text{S}}}_{{\text{n}}}^{({\text{I}})}$, synaptic gatings are determined by:

4 $$\begin{array}{c}\frac{{{\text{dS}}}_{{\text{n}}}^{({\text{E}})}({\text{t}})}{{\text{dt}}}=-\frac{{{\text{S}}}_{{\text{n}}}^{\left({\text{E}}\right)}}{{\uptau }_{{\text{NMDA}}}}+\left(1-{{\text{S}}}_{{\text{n}}}^{\left({\text{E}}\right)}\right)\upgamma {{\text{r}}}_{{\text{n}}}^{\left({\text{E}}\right)}+\mathrm{\sigma g }{{\text{v}}}_{{\text{n}}}({\text{t}})\\ \frac{{{\text{dS}}}_{{\text{n}}}^{\left({\text{I}}\right)}({\text{t}})}{{\text{dt}}}=-\frac{{{\text{S}}}_{{\text{n}}}^{\left({\text{I}}\right)}}{{\uptau }_{{\text{GABA}}}}+{{\text{r}}}_{{\text{n}}}^{\left({\text{I}}\right)}+\mathrm{\sigma g }{{\text{v}}}_{{\text{n}}}({\text{t}})\end{array}$$

The decay constants for glutamatergic activity and GABAergic activity are given by ${\uptau }_{{\text{NMDA}}}=0.1\ {\text{s}}$ and ${\uptau }_{{\text{GABA}}}=0.01\ {\text{s}}$, respectively, the excitatory kinetic parameter constant is $\upgamma =0.641$, and the uncorrelated gaussian noise has amplitude $\mathrm{\sigma g}=0.01\ {\text{nA}}$. Given that the observable of the model that is meant to be fitted on the empirical BOLD signals, we applied a transformation from the simulated firing activity of each excitatory pool to BOLD fMRI signals using the Balloon-Windkessel (BK) model (Fig. 1D), as previously proposed by Deco et al. (2018) [25].

We employed the FastDMF implementation (https://gitlab.com/concog/fastdmf) to overcome computational limitations when calibrating the FIC parameter which stabilize firing rates of excitatory pools, as well as when optimizing the parameters that fit the empirical functional connectivity data of each group. This is a computationally efficient DMF implementation including the estimation of the FIC parameter based on the structural connectivity, which leverages Bayesian optimization to accelerate model fitting [47] (for more details on optimization see the Supplementary material).

### Data analysis procedures

For each estimation of our variables of interest, we set $\sigma$ as a free parameter and repeated the process 100 times to then take averages of each prediction. The high-performance computing (HPC) cluster used consisted of two nodes, each containing two Intel Xeon 8268 “Cascade Lake” processors and 192 GB RAM running MATLAB (R2021b, Natick, Massachusetts: The MathWorks Inc.) on parallel. Finally, we obtained i) simulated FC matrix outputs that we could compare with empirical FC matrices ii) $\sigma$ values to correlate with demographic variables, and FC matrices by years with disease to brain connectivity changes across disease course (Fig. 1E).

### Statistical analysis

To compare the empirical FC matrices with simulated FC matrices, we used the structural similarity index measure (SSIM). The SSIM metric is defined as $\frac{2{\mu }_{x}{\mu }_{y}+0.01}{{\mu }_{x}^{2}+{\mu }_{y}^{2}+0.01}\frac{2{\sigma }_{x}{\sigma }_{y}+0.03}{{\sigma }_{x}^{2}+{\sigma }_{y}^{2}+0.03}\frac{{\sigma }_{xy}+0.015}{{\sigma }_{x}{\sigma }_{y}+0.015}$, where x and y stand for the two matrices being compared, and the variables ${\mu }_{x}$, ${\mu }_{y}$, ${\sigma }_{x}$, ${\sigma }_{y}$ ${\sigma }_{xy}$ correspond to the local means, standard deviations, and covariances of matrices x and y, respectively. SSIM has the advantage of simultaneously weighting the Euclidean and correlation distances between matrices. The distance (equivalently, dissimilarities) between empirical and simulated FC matrices was computed as 1 – SSIM. To statistically compare associations between model fit parameters and demographic variable (e.g. gender) as well as to compare between databases of different geographic predominance, we employed analysis of covariance (ANCOVA) (α = 0.05) after confirming normality, homogeneity of variance, and random independent samples of the optimal model parameters. To assess statistically significant differences in the variance of the optimal model parameters across databases that could depend on geographic predominance of the population (e.g. HIC vs. Latam), we used the Levene test (α = 0.05).

We employed the BrainNet Viewer [48] to obtain brain network FC representations to correlate positively and negatively with YWD, and used non-parametric Spearman correlation to examine associations between model parameter $\sigma$ and YWD. Thus, this analysis used the model-generated FC to capture the relationship between $\sigma$ and YWD, and use it to forecast the future evolution of inhibition loss in terms of local atrophy.

We used two sample tests (Benjamini–Hochberg FDR-corrected, $\alpha$=0.05) to assess for statistically significant differences between gender on each region (*N* = 4 comparisons) and the goodness of fit of the models (*N* = 5 comparisons). Effect size was calculated using Cohen’s d, with the objective of comparing model performance with different brain atrophy maps as priors.

## Results

### Biophysical model fitting

**Fig. 2:**
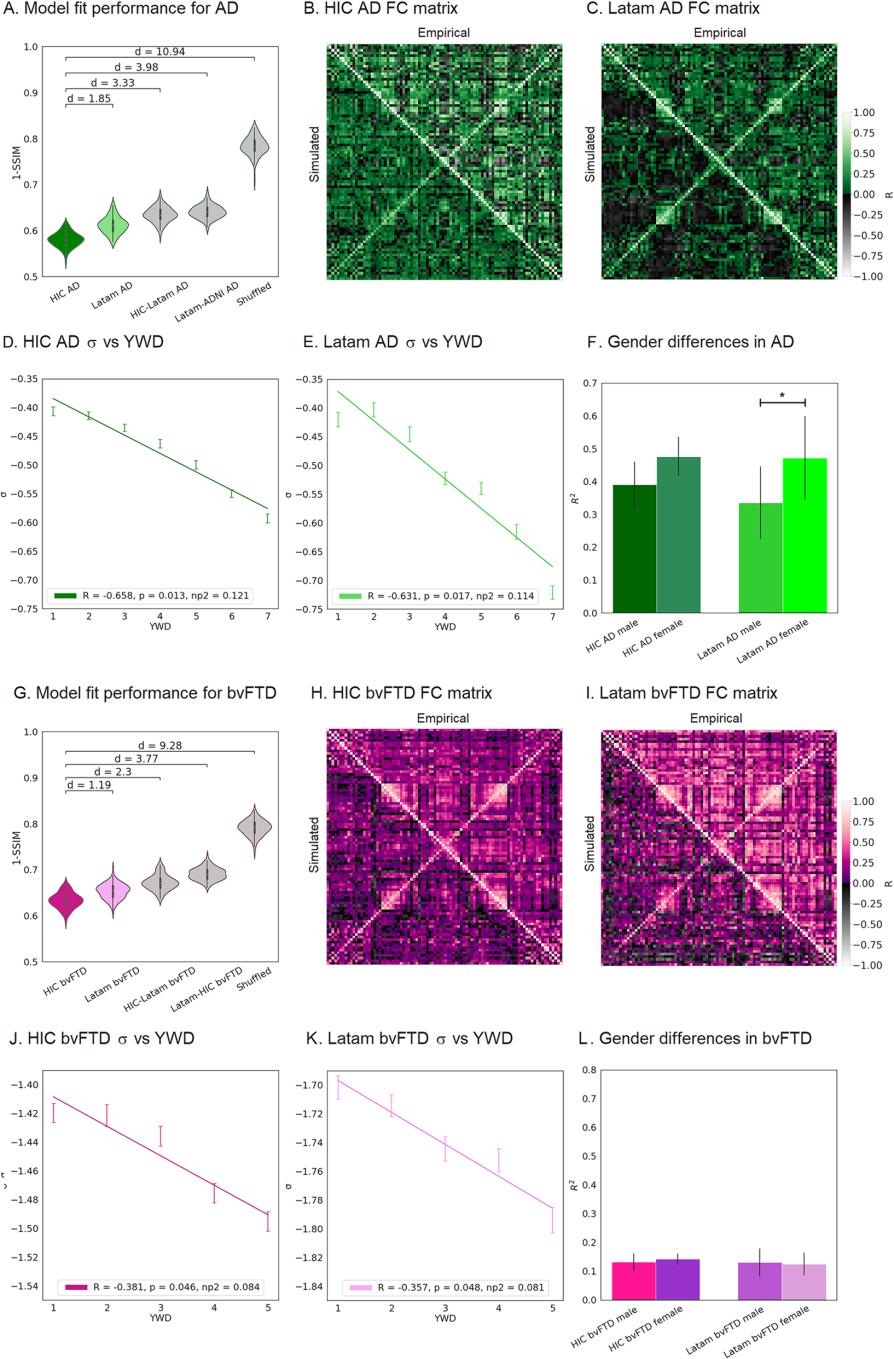
Goodness of fit and relationship between $\upsigma$ and YWD. **A** Model goodness of fit for participants with AD using different atrophy patterns. **B** HIC AD FC matrix with empirical FC matrix above diagonal and simulated FC with optimal parameters below diagonal. **C** Latam AD FC matrix with empirical FC matrix above diagonal and simulated FC with optimal parameters below diagonal. **D** Linear regression of $\sigma$ vs. YWD for HIC AD participants. **E** Linear regression of $\sigma$ vs. YWD for Latam AD participants. **F** Comparison of the coefficient of determination ($\sigma$ vs. YWD) between databases and genders in the AD subgroup, showing significant differences for Latam AD. **G** Model goodness of fit for participants with bvFTD using different atrophy patterns. **H** HIC bvFTD FC with empirical FC matrix above diagonal and simulated FC with optimal parameters below diagonal. **I** Latam bvFTD FC matrix with empirical FC matrix above diagonal and simulated FC with optimal parameters below diagonal. **J** Linear regression of $\sigma$ vs. YWD for HIC Latam bvFTD participants. **K** Linear regression of $\sigma$ vs. YWD for Latam bvFTD participants. **L** Comparison of the coefficient of determination ($\sigma$ vs. YWD) between databases and genders in the bvFTD subgroup, showing no significant differences. SSIM = Structural Similarity Index Measure, d = Cohen’s d, HIC-Latam AD = HIC AD model with Latam AD atrophy, Latam-HIC AD = Latam AD model with HIC AD atrophy, HIC-Latam bvFTD = HIC bvFTD model with Latam bvFTD atrophy, Latam-HIC bvFTD = Latam bvfTD model with HIC bvFTD atrophy. HIC = High Income Country database. Latam = Latin American database. YWD = Years with disease. $\mathrm{\sigma \sigma }$ = Scaling parameter of FIC as a function of local atrophy values. HIC = High Income Country database. Latam = Latin American database. AD: Alzheimer’s disease. bvFTD: behavioral variant frontotemporal dementia

First, we assessed the model fit performance by comparing the empirical FC matrices with the simulated FC matrices by computing 1 – SSIM values. Each simulation was run using the following anatomical priors: subject-specific FC matrices for each group as optimization targets, a DTI structural connectome atlas, and w-score atrophy vectors corresponding to each group and region. The results of model fitting are shown in Fig. 2. The violin plots display 1—SSIM values for AD and bvFTD participants. To assess atrophy specificity, we compared the model fits with the corresponding atrophy for AD patients (Fig. 2A) and bvFTD patients (Fig. 2G) for each region (i.e., Latam and HIC) with respect to switched atrophies between centers (i.e., HIC AD model with Latam AD atrophy (HIC-Latam AD), Latam AD model with HIC AD atrophy (Latam-HIC AD), HIC bvFTD model with Latam bvFTD atrophy (HIC-Latam bvFTD), Latam bvfTD model with HIC bvFTD atrophy (Latam-HIC bvFTD), and also shuffled atrophy vectors, i.e. atrophy vectors with randomly permuted entries. These values were obtained from 100 independent realizations of the Bayesian parameter optimization algorithm built in the FastDMF toolbox.

We found that the best fits were obtained with the correct atrophy vector for each center, and that these models showed statistically significant differences with respect to the shuffled atrophy maps (all *p* < 0.001, FDR-corrected). Moreover, for both AD and bvFTD, the correct atrophy models had statistically significant higher performance with respect to the other atrophy models, with average 1-SSIM = 0.59 for HIC AD and 1-SSIM = 0.61 for HIC Latam, and 1-SSIM = 0.63 for HIC bvFTD and 1-SSIM = 0.65 for Latam bvFTD, compared to switched atrophies with a mean of 1-SSIM = 0.66 for AD and 1-SSIM = 0.68 for bvFTD (all *p* < 0.001, FDR-corrected), and shuffled atrophies with a mean of 1-SSIM = 0.79 for AD and bvFTD (all *p* < 0.001, FDR-corrected), showing that meaningful atrophy patterns improve the fit to the empirical whole-brain FC matrix. Fitting values averaging 1-SSIM≈0.6 are considered good model fits as reported in previous publications on similar models based on different datasets [30, 49]. Comparing fits between HIC datasets (AD and bvFTD) and Latam (AD and bvFTD) participants, we found a large effect size separating HIC AD atrophy models and Latam AD atrophy models (Cohen’s d = 1.85) (Fig. 2A) and a high effect when comparing goodness of fit of HIC bvFTD models vs. Latam bvFTD atrophy models (Cohen’s d = 1.19) (Fig. 2G). To account for the potential effects of different sample sizes and group imbalances between the HIC and Latam databases, we performed a reproducibility analysis. Fitting the HIC model with a sample size group distribution matched with the Latam model yielded generalizable results (see Figure S1 of the Supplementary material). We obtained similar overall fitting performances on the HIC subsample (1-SSIM≈0.63) when compared to the HIC full sample (1-SSIM≈0.6) (*n.s*.: *p* > 0.05), with negligible differences in performance when modeling different pathologies when comparing both datasets (AD models Cohen’s d = 0.12, bvFTD models Cohen’s d = 0.14) and with similar $\sigma$ FIC parameters (all *p* > 0.05).

### Local inhibition changes and years with disease

We next aimed to test if the parameter $\sigma$ was associated with disease progression via the YWD variable. To this end, we ran simulations with the subjects separated according to YWD (100 iterations each) for the HIC AD model, the Latam AD model, the HIC bvFTD model, and the Latam bvFTD model, and performed linear regressions. For the AD models we found significant associations between $\sigma$ and YWD for HIC AD (*R* = -0.658, *p* = 0.013, np^2^ = 0.121) (Fig. 2D), and for Latam AD (*R* = -0.631, *p* = 0.017, np^2^ = 0.114) (Fig. 2E). The ANCOVA analysis (see Figure S2 of the Supplementary material for a visual representation) showed significant differences between models (F-score = 20.95, *p*-value = 0.024 (FDR corrected), np^2^ = 0.04) and YWD (F-score = 9.53, *p*-value = 0.001 (FDR corrected), np^2^ = 0.36) for the gender variable for the Latam participants with AD only. Notably, the linear regression models showed that women had a higher rate of progression (*R* = -0.687, *p*-value = 0.01, np^2^ = 0.169, R^2^ = 0.471) than men (*R* = -0.579, *p*-value = 0.024, np^2^ = 0.124, R^2^ = 0.335) (Fig. 2F). For the bvFTD models we found significant associations between $\sigma$ and YWD for both HIC bvFTD (*R* = -0.381, *p* = 0.046, np^2^ = 0.084) (Fig. 2 J), and for Latam bvFTD (*R* = -0.357, *p* = 0.048, np^2^ = 0.081) (Fig. 2K). The models for the bvFTD participants did not evidence significant differences in terms of gender (Fig. 2L).

### Local inhibition changes by patient group, origin, and gender

**Fig. 3:**
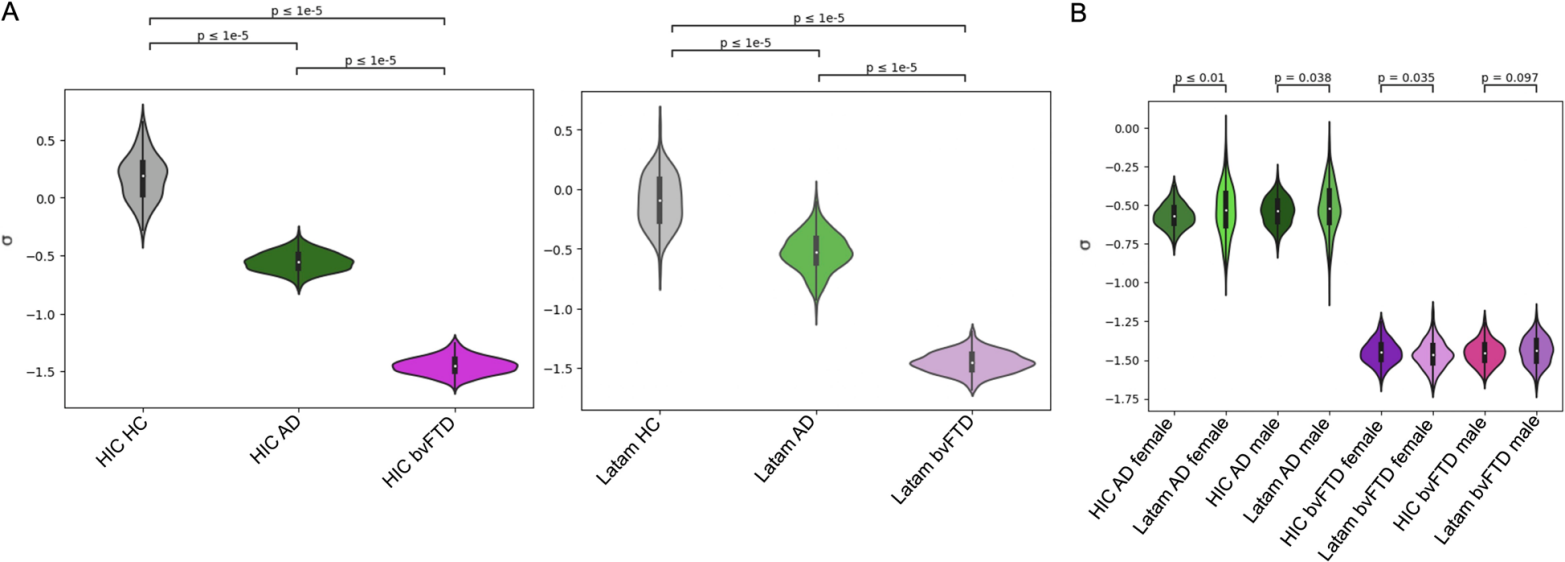
Overview of the optimal model parameter $\upsigma$, related to the effect of atrophy on feedback inhibitory current. **A** $\upsigma$ for the different subgroups, both for HIC (left) and Latam (right). **B** $\upsigma$ separated by geographic origin, patient subgroup (AD and bvFTD) and gender. In both cases, shown *p*-values were obtained using an independent two sample t-test

Next, we investigated the $\sigma$ parameter, linking brain atrophy to local inhibition. The results of this analysis are shown in left and right panels of Fig. 3A for HIC and Latam, respectively. This figure shows that $\sigma$ was approximately zero (i.e. no effect of atrophy on FIC) for the control group, and progressively more negative for AD and bvFTD (i.e. increased reductions in FIC). This was replicated for both HIC and Latam. Figure 3B presents the $\sigma$ values per patient group, cohort, and gender, with significant differences between disease groups (AD vs. bvFTD) and cohorts (Latam vs. HIC) in female participants. Notably, the Latam models showed a statistically significant higher $\sigma$ spread than the HIC models according to a Levene test (*p* < 0.05).

### Biophysical model predictions of disease progression

**Fig. 4:**
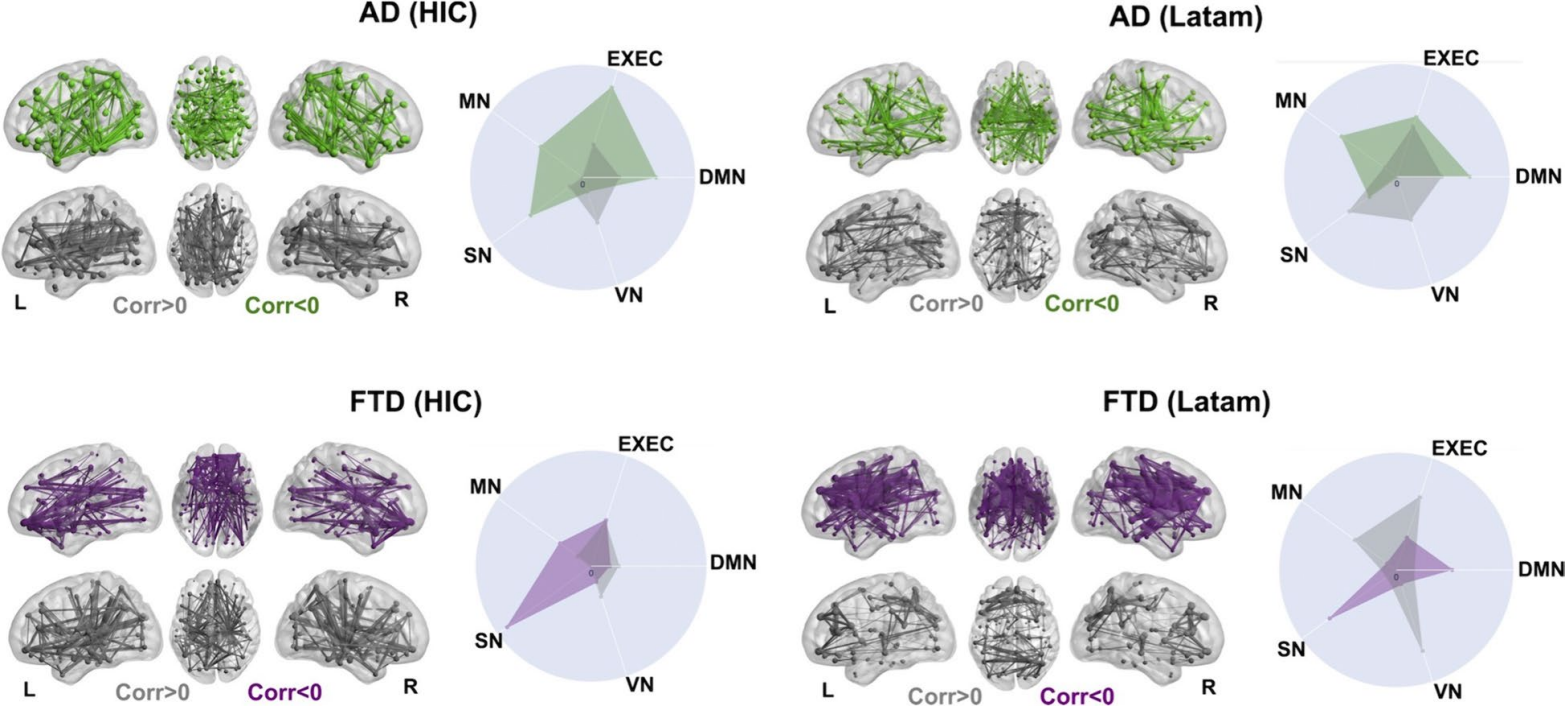
Decreases and increases of FC vs. YWD predicted by the biophysical model. AD from HIC (panel **A**), AD from LAtam (panel **B**), bvFTD from HIC (panel **C**) and bvFTD from Latam (panel **D**). The sagittal anatomical overlays present the top 5% of pairwise FC connections in terms of their negative (green for AD, purple for bvFTD) and positive (grey in both cases) Spearman correlation with YWD. The radar plots indicate a significant overlap of the regions with negative FC changes vs. YWD and DMN/EXEC RSN for AD, and with SN RSN for bvFTD. VN: visual network, SN: salience network, MN: motor network, EXEC: executive control network, DMN: default mode network; FC; functional connectivity, YWD: years with disease; AD: Alzheimer’s disease; bvFTD: behavioral variant frontotemporal dementia

Finally, we assessed how the model predicted FC changes vs. YWD, both for negative correlations (i.e., FC decrease with cumulative YWD) and positive correlations (i.e., FC increase with cumulative YWD). The objective of this analysis was to extrapolate the evolution of FC with years with disease, assisted by the model that was fitted to the empirical data. This was implemented at node level first, and then at the brain network level. For this purpose, we obtained 100 independent runs of the simulation for each of the optimal $\upsigma$ corresponding to the different numbers of YWD. Then, we computed the corresponding FC matrices and obtained the average Spearman correlation between each entry in the FC matrix and the YWD. The results of this analysis are shown in Fig. 4, where the mean Spearman correlation value between pairwise FC and YWD (as predicted by the model) is shown. For the participants with AD in high income countries, in the years with disease analysis, we observed a decrease mostly in lower temporal and parietal FC, and a mild increase in FC in fronto-occipital connections (Fig. 4A, left subpanel). For the participants with AD in the Latin American region, we saw a mild decrease mostly in lower temporal and parietal FC, and a mild increase in FC in fronto-parietal connections (Fig. 4B, left subpanel). For the participants with bvFTD in high income countries, we noticed a decrease mostly in connections stemming from orbitofrontal nodes, and an increase in lower temporal and parietal FC was evidenced (Fig. 4C, left subpanel). Finally, for the Latam participants with bvFTD, a decrease was noticed mostly in connections stemming from the anterior cingulate cortex, with a mild increase in scattered networks comprising occipito-parietal and frontal FC (Fig. 4D, left subpanel).

These patterns of change can be put into correspondence with known resting state networks (RSN). We employed a set of canonical RSN masks from an atlas of functional ROIs averaged in MNI space [50] (VN: visual network, SN: salience network, MN: motor network, EXEC: executive control network, DMN: default mode network). We then computed the average Spearman coefficient (FC vs. YWD) of each node, separately for positive vs. negative FC, and thresholded these vectors to match the number of non-zero entries in the RSN maps parcellated using the AAL atlas. Finally, we computed the overlap between the binarized maps of positive/negative correlation of FC. vs. YWD and each RSN by means of the Jaccard index between the corresponding binary vectors. Results are presented in the radar plots of Fig. 4. While the results for positive associations did not follow a clear trend, we observed a robust association between FC decrease with YWD overlapping with RSN and EXEC networks for AD patients (Fig. 4A and B, right subpanels), while for bvFTD patients this association was present almost exclusively at the SN (Fig. 4B and C, right subpanels).

## Discussion

While animal studies support the role of hyperexcitability in the onset and progression of neurodegenerative diseases, the in vivo assessment of neural excitation/inhibition is difficult in human neuroimaging studies. In this study, we used a biophysical model of whole-brain activity to investigate the potential relationship between alterations in functional neuroimaging data and shifts from normal levels of local inhibitory currents that may underlie increased network hyperactivity in dementia patients. Using this model, we demonstrated loss of local inhibition in AD and FTD patients, with changes specific to anatomical regions presenting neurodegeneration, as determined by the independent analysis of structural brain images. We also found an effect of gender, geographical origin, and years with disease on the local inhibition model parameter, and by simulating its progression we reproduced known results concerning whole-brain functional connectivity changes in dementia patients.

### Biophysical modeling supports a link between neurodegeneration and loss of inhibition

While our model did not directly assess the balance between neural excitation and inhibition, previous research supports the choice of modeling changes in local inhibition in AD and bvFTD patients [9, 11, 18, 20, 21]. In turn, our results provide support for the hypothesis that alterations in neural inhibition modulated by disease-specific atrophy patterns can underlie changes in functional connectivity measured in neurodegeneration. In line with this hypothesis, previous studies reported reduced inhibitory interneuron activity as a potential cause of neural hyperactivity in AD animal models [18, 51]. The resulting network hyperactivity increases the release of soluble amyloid [52, 53], triggering hyperexcitability in the form of epileptiform discharges in patients with AD [53], thus leading to a feedback loop implicating loss of E/I balance, neurodegeneration, and amyloid levels. Crucially, studies in humans showed that amyloid load in AD causes loss of inhibitory GABAergic neural terminals [54], which can displace dynamics towards hyperactivity. Increased amyloid can also trigger neurodegeneration, as shown in a study that found that brain regional atrophy is strongly correlated with amyloid load in early AD [55]. A recent study assessing brain network E/I imbalances in amnestic MCI evidenced a direct relationship between an hyperexcitability-triggered reduction functional connectivity in networks supporting memory formation and atrophy in brain regions also associated with memory [56]. Overall, our results strengthen the reported links between loss of inhibitory synapses and neurodegeneration.

The balance between neural excitation and inhibition can be estimated from resting state MEG and/or EEG data by computing the aperiodic exponent of the signal. This method was validated with simulated local field potentials [24], and applied to AD patients by van Nifterick and colleagues [20], reporting signs of hyperexcitability in demented patients with AD but not in early-stage patients, which is fully consistent with our findings. Our approach is complementary to that of van Nifterick et al., as it is based on a different imaging neuroimaging modality. Moreover, the use of fMRI presents distinct advantages over approaches based on scalp EEG. The high spatial resolution of fMRI facilitates the formulation of a whole-brain model without need of source localization. Data measured using other techniques with equal or superior high spatial resolution can be aligned to the functional MRI data and then incorporated as anatomical priors to constrain the model parameters [57]. Here we followed this approach using brain atrophy measured using structural MRI, but previous models have incorporated spatial maps of receptor density [25], tau and Aβ deposition maps [8] measured with PET.

### Group-specific relationship between loss of neural inhibition and years with disease

Interestingly, we found that averaged subject group-specific atrophy patterns increased model fit in the case of participants with AD, with a lower fit performance for participants with bvFTD. Moreover, using atrophy maps from each dataset and condition allowed us to evaluate whether the observed excitation-inhibition abnormalities also were disease-specific. While the direction of FIC change was the same in AD as in bvFTD, the increasing tendency to hyperexcitability with increasing YWD presented a larger slope for AD compared to bvFTD. The hyperexcitability phenotype is less frequent in bvFTD [16, 17], which might contribute to explain these results.

### Inclusion of disease-specific atrophy maps

Differences in neural cytoarchitecture, neurotransmitters, and ion channel variability across the brain results in functional heterogeneity, including in the predominance of excitation/inhibition [58]. Hence, it is reasonable to expect that neurodegeneration indexed by local atrophy, associated to proteinopathy load [59] and potentially to neural hyperactivity [14] may improve whole-brain multimodal models [30], which was confirmed by our results. Previous computational efforts also attempted to characterize the mechanisms underlying altered large-scale activity and functional connectivity in neurodegeneration and dementia [7–10]. For instance, fMRI resting-state connectivity alterations in AD were reproduced by manipulating brain dynamics through Hopf bifurcation parameters [7, 30], while other studies included anatomical priors related to protein deposition [8]. Moreover, such models were used simulate the presence of neural hyperactivity by using amyloid PET connectomes as input to generate EEG patterns matching empirical signals [9]. Our work presented certain advances over these previous studies, such as the inclusion of disease-specific anatomical priors and the analysis of the impact of sample variability in model fitting performance.

### Effect of demographic and clinical variables on loss of local inhibition

Neurodegeneration phenotypes may differ in HIC compared to Latam regions due to varied social, cultural, and geographic contexts [3–5]. Multiple factors including genetic [3, 60, 61], cognitive [62, 63] and brain structural and functional network features [63, 64], together with socioeconomic disparities can induce heterogeneous presentations of AD and bvFTD [31, 32]. Our approach was able to capture geographic heterogeneity generating differences in model performance. The variability of the parameter linking loss of inhibition to brain atrophy was significantly higher in Latam models, impacting in model fit performance for this population compared to the HIC models. Nevertheless, the Latam models employing geographic-specific atrophy performed better than those based on randomized atrophy patterns. These results highlight the robustness of our model to withstand neurodegeneration phenotype heterogeneity.

Concerning the correlation between YWD and the parameter linking atrophy to hyperactivation, it is known that the GABAergic system presents significant changes in the course of ageing and AD [27, 29]. The loss of inhibitory GABAergic interneurons resulting in network hyperactivity may be a key driver of neurodegeneration in AD by stimulating the spread of amyloid and tau pathology, which in turn promotes excitotoxicity and cell death [28]. This is consistent with our finding that the local inhibition parameter was significantly associated with years of disease, particularly for AD.

Notably, the patient groups did not have a homogenous temporal trajectory, with Latam subjects experimenting a higher rate of change. Moreover, the rate of progression was higher in women participants with AD than in men with AD. Anatomical MRI studies in MCI and AD subjects show that atrophy rates are faster than those in men [65, 66]. Indeed, divergent changes in brain structure were evidenced in quantitative proteomic studies [67], showing that women have more alterations in white matter and mitochondrial proteomes that result in more rapid neurodegeneration. Brain structure differences may be exacerbated by gender inequality impacting mental health [67], where exposure to adverse environments can decrease dendritic branching and synapse formation impacting cognitive reserve [68]. In the case of the bvFTD participants, such differences were not present. This may be explained by the higher prevalence of bvFTD in men and the typically later age of onset in women [69]. Third, women have a tendency to suffer from language impairments associated with the less frequent presentation of primary progressive aphasia [69], adding heterogeneity to this group in its atrophy patterns.

The comparison of patient samples from different geographic regions requires the analysis of multi-center data acquired with different MRI scanners, potentially obscuring significant differences. However, our analysis was capable of revealing differences in the FIC between groups as well as an association with years with disease, in spite of the variability that could have been introduced by the different scanning sites. On the other hand, while some of the results presented higher spread in the Latam group compared to the HIC group, this was not universally observed for all comparisons, suggesting the absence of a systematic bias due to scanner and sequence heterogeneity.

The present analysis could only attribute differences between the Latam and HIC samples to their geographical origin. To address this non-specificty, future studies should replicate our findings using better characterized samples, including data on environmental exposure, social determinants of health, socioeconomic status, risk factors and genetic diversity, which are factors implicated in the disparity between Latam and HIC [70–75].

### Consistency with previous reports of hypoactivation in advanced AD patients

While increased task-evoked activation is characteristic of prodromal AD, some studies support the opposite result in MCI and AD patients with dementia, resulting in an inverse U-shape for brain activation [76]. In contrast, our results show decreased neural inhibition in AD patients, which could translate to facilitated activation. However, MCI patients may also exhibit hyperactivation, which also predicts the progression to dementia [77]. The diffuse boundary at the hyper- to hypoactivation transition could be related to variability in tau and amyloid-beta (Aβ) burden. Indeed, biophysical models applied to MEG data suggest that changes in excitatory/inhibitory time constants are linked to deposition of tau and Aβ [8, 13], and a study of healthy aging individuals found a quadratic relationship between Aβ burden and hyperactivation [78]. As this information was not available for all the participants in our sample, it is not possible to assert that our finding decreased inhibition contradicts previous reports of hypoactivation.

Another important point concerns the difference between hyper/hypoactivation of task-evoked vs. intrinsic activity. As discussed above, several studies point towards hyperexcitable spontaneous activity in AD patients. Converging evidence is provided by animal studies [18], studies of cortical excitability assessed with TMS in humans [19], and by the investigation of the excitation/inhibition ratio as estimated from spontaneous MEG activity using data-driven analyses [20]. Task-evoked hyper- or hypoactivation could depend on multiple factors such as altered top–bottom attentional processes, cognitive impairment due to cortical atrophy, and neurovascular coupling, among others [79]. Importantly, these factors do not necessarily reflect changes in local excitation or inhibition, such as those implicated in the reduction of the FIC shown by our model-based approach.

### Modeling changes in functional connectivity during disease progression

Whole-brain modeling results reflected data-driven disease-specific profiles of functional brain connectivity loss [80]. Previous research comparing fMRI functional connectivity node affectation between AD and bvFTD has shown that bvFTD subjects have reduced nodal strength in the frontoinsular area, a relatively focal altered functional connectivity between key components of the SN that are affected in bvFTD while a functional connectivity breakdown in posterior brain nodes, particularly in the parietal lobe, characterizes AD [81]. Posterior nodes are critical components of the DMN, which is a network associated with autobiographic memory associated with specific AD-affected nodes [82]. Alongside DMN alterations that are hallmark in AD [83], we found EXEC network impairments that have been also reported in early AD [84]. Moreover, we found that AD presented posterior resting state network affectation such as in DMN, with an underlying anatomical structure atrophy, while on the other hand bvFTD showed anterior resting state network affectations within the SN, also with underlying atrophy in associated brain structures [85].

Our model predicted both positive and negative correlations of FC with YWD as a consequence of loss of local inhibition. Compensatory mechanisms of the neurodegenerative process resulting in hyperconnectivity are present even at early disease stages [86], and may vary during disease progression [87]. However, the compensatory hypothesis is currently under debate, pointing to neuronal network hyperactivity due to loss of inhibitory synapses as a driver of neurodegeneration rather than as a compensatory effect [29]. Resting state fMRI alone may not be sufficient to address the possibility of compensatory mechanisms, which could require a more direct connection with brain activity underlying the cognitive functions that are compromised in patients with dementia [88].

### Strengths and limitations

Our study presents some limitations and considerations for further research. First, the sample size, though limited, was comparable and in some cases even higher to similar previous work in the literature [8, 13, 25, 30], and was sufficient to obtain robust and specific results when modeling heterogeneous samples. However, some of the reported *p*-values (mainly those related to the association between changes in FIC and demographic variables) were above the threshold of 0.005, prompting the need for an independent replication of these results. Second, while sample imbalances across regions may tend to bias the results, our analysis employing a matched HIC sample rules out that possibility. Third, our distinction between HIC and Latam samples may not cover all possible sociodemographic nuances, requiring stricter demographic control in future studies. Fourth, dementia diagnosis typically relies on clinical criteria, but biomarkers like amyloid-β and tau proteins measured by PET or plasma are also used [89]. However, clinical criteria is valid for research [90, 91], and PET/plasma biomarkers have limited availability and low cost-effectiveness [4], and they do not provide a conclusive diagnosis nor discriminate well between FTD variants [92]. Plasma biomarkers hold promise but lack systematic validation in diverse populations [93]. Future research should combine clinical and biomarker criteria to model whole-brain dynamics, and use metabolic maps of tau and amyloid deposition priors to improve the fit of whole-brain modeling, considering the link between these proteins and changes in neural excitability [8, 13]. Fifth, other metrics of heterogeneity, such as genetics/admixture features and other measures of disease progression may capture brain phenotype diversity in a more comprehensive way. Sixth, our atrophy measures were averaged across groups, sharing this limitation with other work in the literature [30]. However, our model allows the individualization of input parameters as distinct atrophy vectors, opening the possibility of studying the single-subject progression of neurodegeneration. A related limitation is the use of an averaged reference connectome and approximation of zero-lag interaction between cortical regions. The conversion from firing rates to hemodynamic activity via the Balloon-Windkessel model blurs the input signals, integrating over the slow time scale characteristic of the vascular response, and thus filtering our fast signal variability that could emerge due to variability in the propagation velocity [94, 95]. On the other hand, the lack of good quality DTI for all participants led us to employ an average connectome, a frequent approximation in whole-brain modeling studies [7–9, 23, 30]. The loss of white matter integrity in AD occurs in patterns that are generally independent of grey matter atrophy [96], which modulated the FIC in our model, suggesting that connectome differences were not the primary driver of our findings. As high-quality DTI connectomes become increasingly available, this limitation could be overcome through personalized virtual brain models, which hold promise for addressing individual variability in various neurological conditions, including neurodegenerative diseases [10, 21, 23, 97–103]. Finally, while fMRI provides neurobiological information, it falls short of giving direct neurophysiological information as other techniques that quantify electrical activity in the brain. Biophysical models have been used in past studies for the indirect inference of neurophysiological information from BOLD signals [104, 105], and our study constitutes evidence that informing fMRI data with models can indeed assist in the mechanistic interpretation of neuroimaging recordings acquired from AD and bvFTD patient groups. However, future studies should employ similar models including EEG or MEG modalities to obtain a more direct proxy of hyperexcitability, which would represent an important step towards the validation of fMRI studies.

## Conclusions

In conclusion, a whole-brain model of AD and bvFTD was developed accounting for non-stereotypical and heterogeneous samples. The proposed model of the pathophysiological mechanism was based on current biological literature pointing to the role of excitation-inhibition alterations as the underpinning of neurodegenerative diseases and their progression. Our model was robust in fitting functional connectivity pattern affectation across years with disease, with results that were specific to overall atrophy patterns and also to geographic and gender differences across subjects, which may contribute to gain a deeper understanding of regional heterogeneity in dementia subtypes.

### Supplementary Information

**Supplementary Material 1.**

## Data Availability

The dataset supporting the conclusions of this article is available in an Open Science Foundation available at the following address: https://osf.io/r6dyq/. The Python codes necessary to reproduce the results presented in this article are available at the same repository.

## Abbreviations

AD: Alzheimer’s disease
bvFTD: Behavioral variant frontotemporal dementia
HC: Healthy controls
fMRI: Functional magnetic resonance imaging
DTI: Diffusion tractography imaging
E/I: Excitation/Inhibition
Latam: Latin America
LAC: Latin America and the Caribbean
HIC: High income countries
HC: Healthy controls
YWD: Years with disease
FIC: Feedback inhibition control
DMF: Dynamic mean field
NINCDS-ADRDA: National Institute of Neurological and Communicative Diseases and Stroke/Alzheimer’s Disease and Related Disorders Association
