# Supplementary material for: Biophysical models applied to dementia patients reveal links between geographical origin, gender, disease duration, and loss of neural inhibition
Source: Alzheimers Res Ther. 2024 Apr 11;16:79. doi: 10.1186/s13195-024-01449-0 (PMC11008050; doi:10.1186/s13195-024-01449-0)
Supplement: Supplementary file 1 — Supplementary Material 1. [file 13195_2024_1449_MOESM1_ESM.pdf]

Supplementary material

1. **Supplementary data 1. Balanced sample demographic details**
2. **Supplementary data 2. Neuroimaging acquisition and preprocessing details**
3. **Supplementary data 3. DTI data collection and processing**
4. **Supplementary data 4. Bayesian optimization**
5. **Supplementary data 5. Results of the reproducibility analysis.**
6. **Supplementary data 6. Visual representation of the ANCOVA results.**

Supplementary data 1. Balanced sample demographic details

Table S1. Demographic statistical results on the HIC balanced sample

**Variable**		**HCs Latam (n = 57) HIC (n = 57)**	**bvFTD Latam (n = 18) HIC (n = 18)**	**AD Latam (n = 39) HIC (n = 39)**	**Statistics (all groups)**	**Post-hoc comparisons**	
						**Groups**	***p*-value**
Sex (F:M)	Latam	38:19	8:10	26:13	χ^2=3.21, p = 0.21 [a]	bvFTD-AD	n.s [b]
						HCs-bvFTD	n.s [b]
						HCs-AD	n.s [b]
	HIC	37:20	9:9	24:15	χ^2=1.28, p = 0.52 [a]	bvFTD-AD	n.s [b]
						HCs-bvFTD	n.s [b]
						HCs-AD	n.s [b]
	Geographic comparison	χ^2=0.04, p = 0.84 [a]	χ^2=0.11, p = 0.73 [a]	χ^2=0.22, p = 0.63 [a]			
Age (years)	Latam	71.16 (8.22)	66.81 (6.34)	76.22 (7.31)	F = 2.09, p = 0.13 [a],	bvFTD-AD	n.s [c]

					$\eta p^2 = 0.03$	HCs-bvFTD	n.s [c]
						HCs-AD	n.s [c]
	HIC	72.25 (8.14)	69.14 (12.11)	74.77 (7.94)	$F = 2.25$, $p = 0.06$ [a], $\eta p^2 = 0.07$	bvFTD-AD	n.s [c]
						HCs-bvFTD	n.s [c]
						HCs-AD	n.s [c]
	Geographic comparison	*t-score* = 1.51, $p = 0.24$[c], $\eta p^2 = 0.02$	*t-score* = 1.82, $p = 0.22$ [c], $\eta p^2 = 0.03$	*t-score* = 1.13, $p = 0.31$ [c], $\eta p^2 = 0.02$			
Years of education	Latam	13.15 (5.15)	16.97 (5.28)	12.54 (7.81)	$F = 2.43$, $p = 0.06$ [a], $\eta p^2 = 0.07$	bvFTD-AD	n.s [c]
						HCs-bvFTD	n.s [c]
						HCs-AD	n.s [c]
	HIC	15.25 (5.36)	14.36 (3.72)	14.02 (4.35)	$F = 3.15$, $p = 0.08$ [a], $\eta p^2 = 0.07$	bvFTD-AD	n.s [c]
						HCs-bvFTD	n.s [c]
						HCs-AD	n.s [c]
	Geographic comparison	*t-score* = 1.56, $p = 0.17$[c], $\eta p^2 = 0.02$	*t-score* = 1.33, $p = 0.22$[c], $\eta p^2 = 0.02$	*t-score* = 0.99, $p = 0.39$[c], $\eta p^2 = 0.01$			

Results are presented as mean (SD). Demographic data was assessed through ANOVAs –except for sex, which was analyzed via Pearson's chi-squared (χ^2) test. Effects sizes were calculated through partial eta squared (ηp^2). HCs: healthy controls, bvFTD: behavioral variant of fronto-temporal dementia, AD: Alzheimer's disease. US: Unmatched sample. MS: Matched sample.

[a] *p*-values calculated via independent measures ANOVA.

[b] *p*-values calculated via chi-squared test (χ2).

[c] *p*-values calculated via unpaired t-test.

Supplementary data 2. Neuroimaging acquisition and preprocessing details

Image acquisition parameters are included in Table S2. MRI cortical thickness metrics and volumetric estimates included voxel-based and surface-based morphometry.[1] The structural volumetric analysis preprocessing included the removal of non-brain tissue, a segmentation of the subcortical white matter (WM) and deep gray matter (GM) volumetric structures (including amygdala, hippocampus, caudate, putamen, and ventricles), and intensity normalization. Finally, we obtained averaged atrophy maps for each group (bvFTD, AD, and HC) and each region in Latam and HIC that were normalized against controls to obtain 90 w-scores (corresponding to the AAL-90 atlas).[2]

For the resting-state protocol, across centers, participants were asked not to think about anything in particular keeping their eyes closed, and to avoid moving artifacts or falling asleep. In each center, we obtained 3D volumetric and 10-minute-long resting-state MRI sequences from all participants. Before preprocessing, the first five volumes of each subject's resting-state session were discarded to ensure a steady state magnetization. The data was then preprocessed using the Data Processing Assistant for Resting-State fMRI (DPARSF V2.3).[3] The images were slice-time corrected (using a reference on the middle slice of each volume) and then aligned to the first scan of the session to correct head movement artifacts. To reduce the effects of motion and physiological artifacts, a total of six head-motion parameters, as well as white matter (WM) and cerebrospinal fluid (CSF) signals, were removed as nuisance variables from the analysis. The WM and CSF masks for this procedure were derived from the tissue segmentation of each subject's T1 scan in the native space. None of the participants showed head movements greater than 3 mm and/or rotations higher than 3°. Finally, images were normalized to the MNI space using the echo-planar imaging (EPI) template from SPM12, smoothed using an 8-mm full-width-at-half-maximum isotropic Gaussian kernel, and bandpass filtered with a frequency range between 0.01–0.1 Hz to correct and remove low-frequency drifts from the MRI scanner. Data was parcellated with the Automated Anatomical Labelling (AAL90).[4] A weighted transformation was performed to control for scanner effects in the data. This process yielded subject-specific timeseries on 90 nodes. Using the pre-processed resting state fMRI time series as input, we captured static and linear associations using Pearson's R static functional connectivity (SFC).[5] Finally, we obtained 90x90 functional connectivity matrices (corresponding to the AAL-90 atlas) per participant.

Table S2. Specific neuroimaging parameters per center

	Parameters
Argentina (center 1)	3-T Phillips scanner with a standard head coil, the whole-brain T1-rapid anatomical 3D gradient echo volumes were acquired parallel to the plane connecting the anterior and posterior commissures, with the following parameters: repetition time (TR) = 8300 ms; echo time (TE) = 3800 ms; flip angle = 8º; 160 slices, matrix dimension = 224 x 224 x 160; voxel size = 1 mm x 1 mm x 1 mm. Also, functional spin echo volumes, parallel to the anterior-posterior commissures, covering the whole brain, were sequentially and ascendingly acquired with the following parameters: TR = 2640 ms; TE = 30 ms; flip angle = 90º; 49 slices, matrix dimension = 80 x 80 x 49; voxel size in plane = 3 mm x 3 mm x 3 mm; slice thickness = 3 mm; sequence duration = 10 minutes; number of volumes = 220.
Chile (center 2)	Using a 3-T Siemens Skyra scanner with a standard head coil, we acquired whole-brain T1-rapid gradient echo volumes, parallel to the plane connecting the anterior and posterior commissures, with the following parameters: repetition time (TR) = 1700 ms; echo time (TE) = 2000 ms; flip angle = 8º; 208 slices, matrix dimension = 224 x 224 x 208; voxel size = 1 mm x 1 mm x 1 mm. On the other hand, functional EP2D-BOLD pulse sequences, parallel to the anterior-posterior commissures, covering the whole brain, were acquired sequentially intercalating pair-ascending first with the following parameters fMRI parameters: TR = 2660 ms; TE = 30 ms; flip angle = 90º; 46 slices, matrix dimension = 76 x 76 x 46; voxel size in plane = 3 mm x 3 mm x 3 mm; slice thickness = 3 mm; sequence duration = 13.3 minutes; number of volumes = 300.
Colombia (center 2)	Using a 3-T Siemens Skyra scanner with a standard head coil, we acquired whole-brain T1-rapid gradient echo volumes, parallel to the plane connecting the anterior and posterior commissures, with the following parameters: repetition time (TR) = 2400 ms; echo time (TE) = 2000 ms; flip angle = 8º; 192 slices, matrix dimension = 256 x 256 x 192; voxel size = 1 mm x 1 mm x 1 mm. Finally, functional EP2D-BOLD pulse sequences, parallel to the anterior-posterior commissures, covering the whole brain, were acquired sequentially intercalating pair-ascending first with the following parameters fMRI parameters: TR = 2660 ms; TE = 30 ms; flip angle = 90º; 46 slices, matrix dimension = 76 x 76 x 46; voxel size in plane = 3 mm x 3 mm x 3 mm; slice thickness = 3 mm; sequence duration = 10.5 minutes; number of volumes = 240.
HIC LONI (Participants with bvFTD)	This sample contained acquisitions from 3T MRI scanners. For T1-weighted images MPRAGE sequences with the following parameters were used: repetition time (TR) = 2300 ms; echo time (TE) = 2.98 ms; flip angle = 25º; 160 slices, matrix dimension = 240 x 256 x 160; voxel size = 1 mm x 1 mm x 1 mm. For fMRI, sequence was EPI-BOLD, the field of view was 220x220x163mm, TE = 30ms, TR=3000ms, flip angle=90º, acquisition time = 10:00 minutes, 2X accelerated (even/odd interleave), P>>A phase encoding.

HIC ADNI (Participants with AD)	This sample contained acquisitions from 3T MRI scanners. For T1-weighted images MPRAGE sequences with the following parameters were used: repetition time (TR) = 6800 ms; echo time (TE) = 3.1 ms; flip angle = 25º; 160 slices, field of view (FOV) of 260-270mm, voxel size = 1 mm x 1 mm x 1 mm. For fMRI, sequence was EPI-BOLD, the field of view was 220x220x163mm, TE = 30ms, TR=3000ms, flip angle=90º, acquisition time = 10:00 minutes, 2X accelerated (even/odd interleave), P>>A phase encoding.

Supplementary data 3. DTI data collection and processing

The structural connectome was obtained applying diffusion tensor imaging (DTI) to diffusion weighted imaging (DWI) recordings from 16 healthy right-handed participants (11 men and 5 women, mean age: 24.75 ± 2.54 years) recruited online at Aarhus University, Denmark. Subjects with psychiatric or neurological disorders (or a history thereof) were excluded from participation. The MRI data (structural MRI, DTI) were recorded in a single session on a 3 T S Skyra scanner. The following parameters were used for the structural MRI T1 scan: voxel size of 1 mm^3; reconstructed matrix size 256 × 256; echo time (TE) of 3.8 ms and repetition time (TR) of 2300 ms. DWI data were collected using the following parameters: TR = 9000 ms, TE = 84 ms, flip angle = 90°, reconstructed matrix size of 106 × 106, voxel size of 1.98 × 1.98 mm with slice thickness of 2 mm and a bandwidth of 1745 Hz/Px. Furthermore, the data were recorded with 62 optimal nonlinear diffusion gradient directions at b = 1500 s/mm^2. Approximately one non-diffusion weighted image (b = 0) per 10 diffusion-weighted images was acquired. Additionally, the DTI images were recorded with different phase encoding directions. One set was collected applying anterior to posterior phase encoding direction and the second one was acquired in the opposite direction. The AAL template was used to parcellate the entire brain into 90 regions (76 cortical regions and 14 subcortical regions). The parcellation contained 45 regions in each hemisphere. To co-register the EPI image to the T1-weighted structural image, the linear registration tool from the FSL toolbox (www.fmrib.ox.ac.uk/fsl, FMRIB, Oxford)[6] was employed. The T1-weighted images were co-registered to the T1 template of ICBM152 in MNI space. The resulting transformations were concatenated, inverted and further applied to warp the AAL template from MNI space to the EPI native space, where the discrete labeling values were preserved by applying nearest-neighbor interpolation. SC networks were constructed following a three-step process. First, the regions of the whole-brain network were defined using the AAL template. Second, the connections between nodes in the whole-brain network (i.e., edges) were estimated using probabilistic tractography for each participant. Third, results were averaged across participants.

Data preprocessing was performed using FSL diffusion toolbox (Fdt) with default parameters. Following this preprocessing, the local probability distributions of fiber directions were estimated at each voxel[6]. The probtrackx tool in Fdt was used to provide automatic estimation of crossing fibers within each voxel, which has been shown to significantly improve the tracking sensitivity of non-dominant fiber populations in the human brain[7]. The connectivity probability from a seed voxel *i* to another voxel *j* was defined by the proportion of fibers passing through voxel *i* that reached voxel *j* (sampling of 5000 streamlines per voxel[7]. All the voxels in each AAL parcel were seeded (i.e. gray and white matter voxels were considered). This was extended from the voxel level to the region level, i.e. in a parcel consisting of n voxels, 5000 × n fibers were sampled. The connectivity probability P_{ij} from region *i* to region *j* was calculated as the number of sampled fibers in region *i* that connected the two regions, divided by 5000 × n, where n represents the number of voxels in region *i*. The resulting SC matrices were thresholded at 0.1% (i.e. a minimum of five streamlines).

Due to the dependence of tractography on the seeding location, the probability from *i* to *j* was not necessarily equivalent to that from *j* to *i*. However, these two probabilities were highly correlated across the brain for all participants ($r > 0.70$, $p < 10^{-50}$). As the directionality of connections cannot be determined using diffusion

MRI, the unidirectional connectivity probability P_{ij} between regions *i* and *j* was defined by averaging these two connectivity probabilities. This unidirectional connectivity was considered a measure of SC between the two areas, with $C_{ij} = C_{ji}$. The regional connectivity probability was calculated using in-house Perl scripts. For both phase encoding directions, 90 × 90 symmetric weighted networks were constructed based on the AAL parcellation, and normalized by the number of voxels in each AAL region, thus representing the SC network organization of the brain of each participant. Finally, the data was averaged across participants.

Supplementary data 4. Bayesian optimization

Bayesian Optimization was implemented in Matlab. An expected-improvement acquisition function was used to optimize the DMF model parameters[8]. The optimization was run assuming a stochastic objective function, letting the algorithm randomly select the initial conditions for each simulation

Supplementary data 5. Results of the reproducibility analysis.

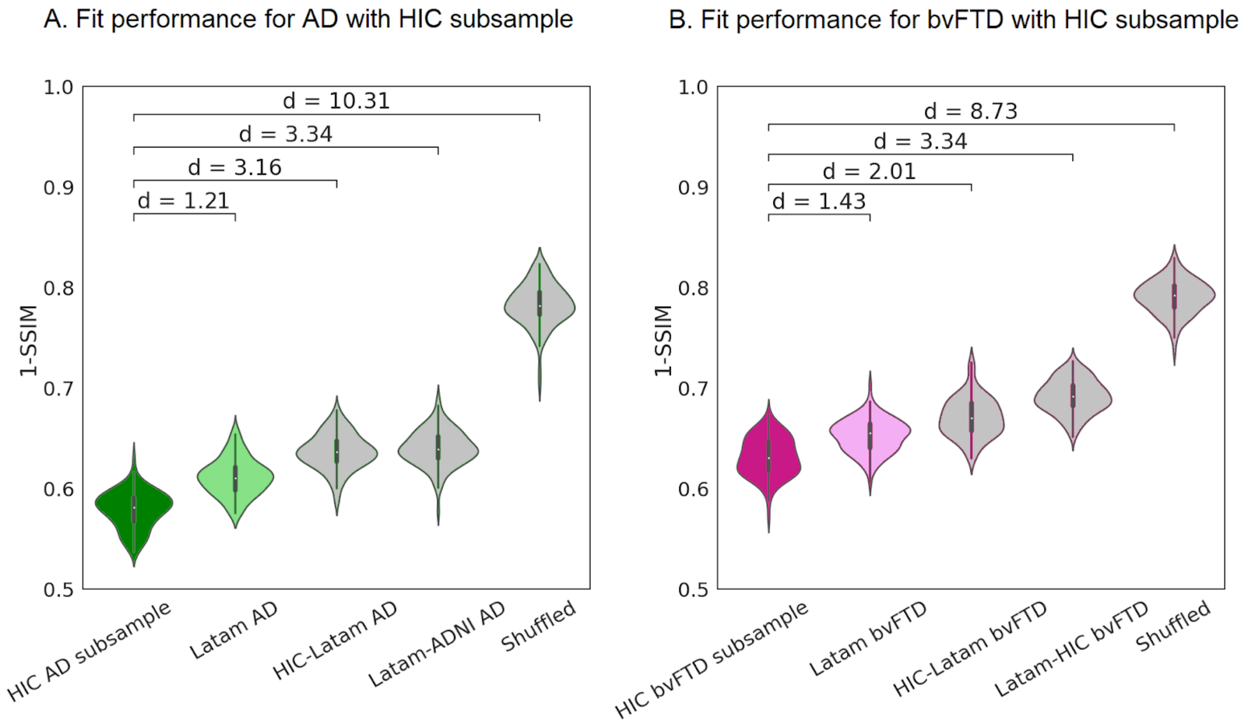

Figure S1. Replication of panels A and G of Fig. 2, but using a subsample of the HIC group matching the Latam group sample size, and also balanced in terms of demographic variables.

Supplementary data 6. Visual representation of the ANCOVA results.

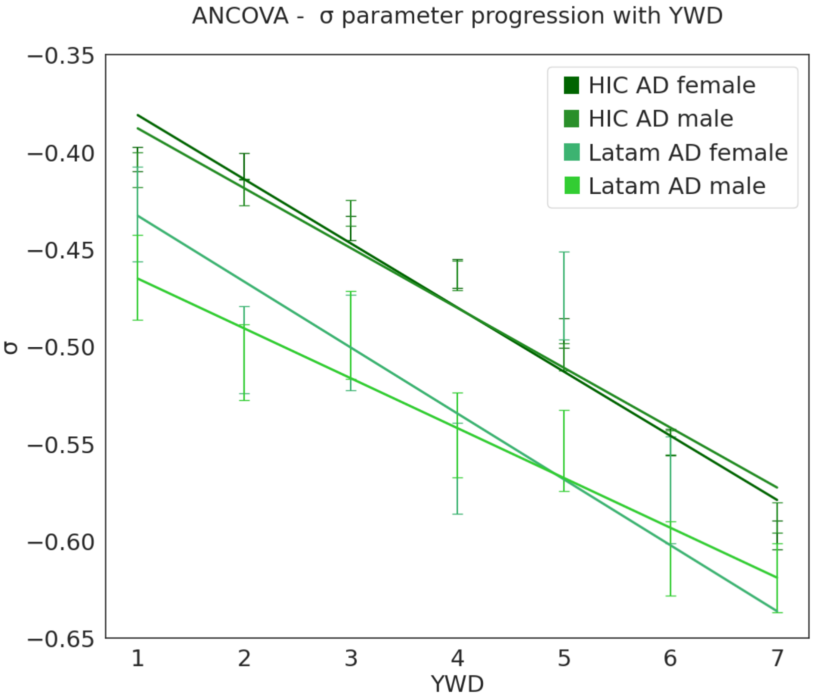

Figure S2. Visual representation of the ANCOVA results. Model parameter vs. years with disease (YWD) fits for all the factor combinations in the analysis (HIC/Latam, male/female), shown for AD patients only, given that the ANCOVA analysis showed significant differences between models for the gender variable in the AD Latam sample.

References

1. Frisoni GB, Fox, N. C., Jack, C. R., Jr, Scheltens, P., & Thompson, P. M. The clinical use of structural MRI in Alzheimer disease. Nature reviews Neurology. 2010;6(2):62-77.

2. Donnelly-Kehoe PA, Pascariello GO, García AM, et al. Robust automated computational approach for classifying frontotemporal neurodegeneration: multimodal/multicenter neuroimaging. Alzheimer's & Dementia: Diagnosis, Assessment & Disease Monitoring. 2019;11(1):588-598.

3. Chao-Gan Y, & Yu-Feng, Z. DPARSF: A MATLAB Toolbox for "Pipeline" Data Analysis of Resting-State fMRI. Frontiers in systems neuroscience. 2010;4(13)doi:https://doi.org/10.3389/fnsys.2010.00013

4. Tzourio-Mazoyer N, Landeau B, Papathanassiou D, et al. Automated anatomical labeling of activations in SPM using a macroscopic anatomical parcellation of the MNI MRI single-subject brain. Neuroimage. 2002;15:273-289. doi:10.1006/nimg.2001.0978

5. Zhou J, Greicius MD, Gennatas ED, et al. Divergent network connectivity changes in behavioural variant frontotemporal dementia and Alzheimer's disease. Brain. May 2010;133(Pt 5):1352-67. doi:10.1093/brain/awq0756.

6. Jenkinson, M., Bannister, P., Brady, M., & Smith, S. (2002). Improved optimization for the robust and accurate linear registration and motion correction of brain images. Neuroimage, 17(2), 825-841.q scs

7. Behrens, T. E., Berg, H. J., Jbabdi, S., Rushworth, M. F., & Woolrich, M. W. (2007). Probabilistic diffusion tractography with multiple fibre orientations: What can we gain?. neuroimage, 34(1), 144-155.

8. Shahriari, B., Swersky, K., Wang, Z., Adams, R. P., & De Freitas, N. (2015). Taking the human out of the loop: A review of Bayesian optimization. Proceedings of the IEEE, 104(1), 148-175.
